# Just Transition on air quality governance: a case study of heavy-duty diesel truck protests in Taiwan

**DOI:** 10.1007/s11625-023-01311-6

**Published:** 2023-05-02

**Authors:** David Walther, Kuei-Tien Chou

**Affiliations:** Risk Society and Policy Research Center (RSPRC), National Taiwan University, Taipei, Taiwan

**Keywords:** Just Transition, Air quality, Environmental justice, Yellow Vests movement, License-leasing drivers, Low-carbon transition

## Abstract

Just Transitions are gaining attention in environmental research, and most studies have focused on climate change; however, the insights from this work may be usefully applied to the rarely discussed area in just transition studies. This article uses traditional dimensions of environmental and social justice, such as distributive, procedural, recognition, and restorative justice, to understand why heavy-duty diesel truck drivers fought back against stricter air pollution regulations while demanding destigmatization. The protest resulted in policy failure, and Taiwan’s transition to cleaner, newer diesel trucks were halted. This study finds that the key social contextual factor in Taiwan’s transportation industry was the labor relations of license-leasing. The drivers’ protest began with a lack of procedural justice, and communication occurred only after the law was passed. There was insufficient regard for procedural justice, and although the drivers were concerned, the new rule would significantly impact their right to work and life. Furthermore, the drivers felt disrespected and even carried the stigma of creating environmental pollution. The article assumes that the results should be different if the governance mechanism can handle the key factor in a social context and make appropriate arrangements for the four dimensions of Just Transition. This argument may be relevant for other countries looking to transition from older diesel vehicles to cleaner vehicles through Just Transition.

## Introduction

In November 2018, the world-astounding French “Mouvement des gilets jaunes” (Yellow Vests movement) marked 26 years of sustainable development since the Rio Declaration on Environment and Development, reaching the conflict point of Just Transition. Remarkably, in July of the same year, Taiwan tightened pollution emission regulations on vehicles older than 10 years. The plan is to phase out 86,386 heavy-duty diesel trucks (HDDTs) in stages I (licensed before June 1993) and II (licensed before June 1999) over 4 years. The National Old Car Self-Help Association (NOCSA), a temporary group of drivers, launched a massive protest in response to this policy. Protesters honked their horns as they drove 500 HDDTs to the Presidential Palace, the administrative center, and the central business district. The scale of the protest stunned the entire island. Furthermore, the “Innocent the Old Cars Protest” (IOC-Protest) indirectly impacted the ruling party’s defeat in that year’s national election. In this context, it is worthwhile to consider the reflections and insights that the IOC-Protest may bring to Taiwan, Asia, and the rest of the world.

In 2017, Taiwan’s government and the public agreed to improve air quality. The Environmental Protection Administration Taiwan (TEPA) revised the Air Pollution Control Act to tighten regulations on vehicles older than 10 years. This triggered the transportation industry to launch the IOC-Protest through NOCSA, claiming “Old cars are innocent; our livelihoods are at stake; do not stigmatize us.” After the IOC-Protest, the ruling party began phasing out older heavy-duty diesel vehicles (HDDVs). They were replaced by an ongoing subsidy to facilitate vehicle replacement. The decision to impose stricter controls on HDDVs older than 10 years sparked the IOC-Protest, prompting the government to halt the process, implying that air quality governance in Taiwan necessitates discourse and debate regarding Just Transition.

This article has two major objectives. First, it analyzes why Just Transition is an important element and force in air quality governance, and then it identifies the aspects of Just Transition that affect environmental policy and governance outcomes. Second, the article explores how Just Transition affects policy and what the actual contents of Just Transition are that stakeholders care about, but policymakers should be aware of. Addressing these issues may help mitigate the effects of Just Transition management failures on air quality and environmental policy implementation. This article describes the case’s background, and then discusses Just Transition frameworks and air quality. Following the introduction of the research structure and method, the case analysis and results are performed in the following order: (1) distributional justice, (2) procedural justice, (3) recognition justice, and (4) restorative justice. Finally, the discussion and conclusion are presented.

## Research background and literature review

### The research background

The anti-air pollution movement in Taiwan began in 2010 with the Movement Against the Kuo-Kuang Petrochemical Industry, and supporters quickly spread throughout Taiwan. As a result, anti-air pollution awareness has grown throughout Taiwan. Following the June 2015 anti-air pollution march in nine counties and cities, biannual national anti-air pollution marches were established, with air pollution becoming the most prominent public issue (Chen and Ho 2017; Walther 2018). Consequently, Taiwan launched a long-term civic epistemology-based anti-air pollution movement, and the region entered a phase where citizens’ demands drive government policies (Jasanoff 2011; Chou 2015). Furthermore, several polls conducted before the 2018 local elections and referendum revealed that air pollution was a major source of public dissatisfaction with the government’s administration (Lin 2018; Lee 2019; Peng 2018; Taichung City Government 2019).

Under pressure from the anti-air pollution movement, the government adopted the Clean Air Action Plan (2015–2020) in August 2015; however, environmental groups and private citizens viewed the plan as passive. Many expected the Democratic Progressive Party (DPP), which was previously more supportive of the environmental movement, to take a more active role in improving air quality. In 2016, the DPP administration expressed its commitment to reducing air pollution by appointing Chan Shun-Kuei, a lawyer with extensive environmental movement experience, as TEPA’s deputy director. Because of the increased channels and influence of civil participation in decision-making, environmental groups can work through the government to push for revisions to the air pollution fee system and an efficient air pollution control plan (the so-called Plan 14 + N). Therefore, Taiwan has entered a golden age of air pollution reduction (O2a; CG2; TEPA 2017). Simultaneously, the TEPA has been working hard to revise the Air Pollution Control Act (hereinafter the new Act). Compared to the original Air Pollution Control Act, the new Act amends 78 articles, significantly improving previous legal deficiencies in air pollution control (O2b; TEPA 2018a, b). Articles 36 and 44 of the new Act lay the groundwork for “stricter control of vehicles manufactured 10 or more years ago.” The second paragraph of Article 36 of the new Act authorizes the competent authorities to impose stricter controls.

The TEPA plans to accelerate the phase out of HDDVs older than 10 years by imposing stricter emission standards and subsidizing replacements. Despite the new Act targeting HDDVs (trucks and buses), HDDTs over 10 years old accounted for 72.8% (stages I–III, licensed before September 2006) in 2016, whereas diesel buses accounted for only 24.9%. There are 86,386 HDDTs (51.8%) in stages I and II, followed by 35,123 (21%) in stage III; therefore, freight transport (trucks) faces more severe impacts than passenger transport (buses). After the draft was passed in December 2017, transportation industry discussions began about 10 or older vehicles being phased out quickly: approximately 86,386 HDDTs and 1 million two-stroke motorcycles would be phased out.

NOCSA was formed gradually over several months and decided to protest in June 2018. Over 500 HDDTs arrived in Taipei at 8:00 a.m. on July 15 from all over Taiwan. The motorcade protested at the Presidential Palace and the Bo’ai Special Zone, which houses many government officials and the Congress. When crossing the 600-m road in front of the Presidential Palace and the Legislative Yuan (Taiwan’s Congress, ROC), protesters held red and white banners reading “INNOCENT THE OLD CARS, OBJECTION TO FORCED PHASE-OUT” and “TODAY I’M FORCED TO REPLACE MY CAR WITH A NEW ONE, TOMORROW THE GOVERNMENT WILL BE REPLACED BY ANOTHER.” Finally, the protesters honked their horns in front of the TEPA, producing a deafening sound (M1a). This incredible march drew extensive media coverage and national attention. TEPA held a quick briefing on July 21 to highlight the relevant support subsidy programs (Lee 2019). However, NOCSA members believed the subsidies were insufficient to compensate for the losses and decided to continue their resistance.

During this time, the authorities did not provide a satisfactory response or disposition to NOCSA. Therefore, on July 28, 2018, 123 drivers in Kaohsiung City petitioned the government (M1a; Liao 2018). Nevertheless, the new version of the Act was passed on the third reading in the provisional session of the Legislative Yuan on June 25, 2018. It was scheduled to be announced for implementation on August 1 (Legislative Yuan 2018). Consequently, the number of protesting vehicles increased to 300 on August 8 in Kaohsiung, and the NOCSA also declared that it would continue to fight across Taiwan using HDDTs. Until this point, there had been no consensus between the two sides.

After August 2017, the issue of IOC-Protests became a major issue in local elections, sparking heated political debate. As the end-of-year local elections approached, the government held an emergency press conference on September 10 to announce the transition to a noncompulsory phase out and continued subsidy increases (Liao 2018; M1b; O2b). However, it was too late, and the IOC-Protest was officially acknowledged as contributing to the ruling party’s landslide defeat in the 2018 local elections (Executive Yuan 2018). In the election, the ruling party lost its second-largest city in Taiwan, where it had been in power for 20 years. Initially, the ruling party ruled over four of the six largest cities. Following the election, this was reduced to two, and the original nine counties and cities ruled were reduced to four. After all, the severe political consequences prompted the government to officially announce, and at least twice, the termination of stricter HDDV controls on December 17, 2018, and March 8, 2019.

According to the International Energy Agency (2021) milestones on the path to net zero, the world should stop selling internal combustion engine cars (cars powered by fossil fuel combustion) in 2035. Carbon emissions from transportation will be reduced by 90.27% by 2050 (relative to 2020). Indeed, transportation activities have contributed significant negative environmental impacts, such as air pollution and carbon emissions (Tsoi et al. 2021). Moreover, Just Transition requires transportation transitions. Governments must also manage incentive policies to ensure a Just Transition (Cazzola et al. 2021). According to Tsoi et al. (2021), transportation transition must adapt to socioecological or lifestyle changes (Brand et al. 2020). They suggested a communication problem and that monetary incentives may not encourage modal shifts. Furthermore, effective policies may be unpopular, threatening the citizen engagement process. Thus, decarbonization policies must be flexible to respond to local changes, and mixed policy instruments are most effective. Due to truck driver shortages, the transition from HDDTs to newer trucks is difficult. For instance, California’s Transportation Emissions to Zero program evaluates labor rights, job loss, job training, and transfer (Brown et al. 2021).

Furthermore, Zabin and MacGillvary (2020) recommended that the state focus its subsidies and other assistance on trucking companies to prevent workers from bearing a disproportionate share of the cost of cleaner trucks. Funding is essential in transport transition for policy components and the provision of subsidies to reinforce the push-and-pull effects that encourage cleaner transport modes (Tsoi et al. 2021). Therefore, the challenge of achieving a sustainable transition to HDDVs is a global one, not just in Taiwan.

### The etymology and framework of Just Transition

As the world moves closer to net-zero (greenhouse gas) emissions and sustainable development, the Climate, Energy, and Environmental (CEE) research community has shown a surge of interest in Just Transition research and practice (McCauley and Heffron 2018). Tony Mazzocchi coined the term “Just Transition” in the 1970s. As environmental sustainability becomes more important in the US, the tightened environmental regulations have resulted in the closure of industries and the threat of job loss for laborers (Atteridge and Strambo 2020). The scope of Just Transition is defined by human interventions (e.g., policies) in the pursuit of a better environment, and the social justice issues resulting from the actions’ consequences (McCauley and Heffron 2018; Eisenberg 2019; Pinker 2020; Trades Union Congress 2008). The IPCC (2021) recently provided a simple definition of Just Transition in the AR6 WGIII glossary:

Just Transition is a set of principles, processes, and practices that ensure that no people, workers, places, sectors, countries, or regions are left behind in transitioning from a high-carbon to a low-carbon economy. It stresses the need for targeted and proactive measures from governments, agencies, and authorities to ensure that any negative social, environmental, or economic impacts of economy-wide transitions are minimised, whilst benefits are maximised for those disproportionally affected.

However, the assessment of the negative social, environmental, and economic impacts that disproportionately affect people requires further clarification (Pai et al. 2020). How can it be treated in a fair manner? What is meant by “just,” especially in sociology, needs to be thoroughly studied because justice is one of the core themes of sociology. According to McCauley and Heffron (2018), the urgency of the low-carbon transition must be met with similar language in justice research when developing new consolidated frameworks to provide long-term analysis and solutions. Among the numerous studies on the subject, some scholars consider that following the genealogy and legacy of environmental and social justice research can further aid Just Transition. Heffron and McCauley (2018) proposed three Just Transition dimensions based on the nature of justice to engage a Just Transition research frame that contends distributional and procedural justice, as well as restorative justice. Based on the framework of Heffron and McCauley (2018), Pai et al. (2020) proposed four forms of justice, which amended recognition justice to the framework to deal with fundamental propositions in environmental and social justice research (in some literature, recognition justice is written as recognitional justice or justice as recognition). It is a valuable framework for dealing with the elementary propositions of Just Transition.

Wang and Lo (2021) reviewed 74 journal articles on Just Transition to identify five Just Transition themes, and Upham et al. (2022) conducted a comprehensive literature review and research. They proposed contributions and connections between energy and environmental justice, equity and transitions, energy democracy and the core research themes among three Just Transition perspectives (distributional, procedural, and recognition justice). Although there are numerous concepts and views on Just Transition (Pinker 2020; Krawchenko and Gordon 2021; Gambhir et al. 2018), the Just Transition frameworks, such as those proposed by Heffron and McCauley (2018), Pai et al. (2020), and Upham et al. (2022), have become sophisticated and should be used more in empirical research.

### The broader concepts of Just Transition and air quality governance

In the climate change era, social justice commitments must be provided under the different cultural and economic conditions of each location to hold the promise of the Paris Agreement, and ignoring Just Transition will impede global climate policy progress (Heffron and McCauley 2022; Wang and Lo 2022; Martin and Islar 2021). Successful policies must be impartial to all members of society, focus on transformational restoration, and provide training and support to those affected by climate policies for them to embrace the transition (Mehling 2018).

In Western capitalist countries, the red (labor) and green (environment) have been at odds for a long time; it is the ideological debate and competitive traditions of Anthropocentrism and Ecocentrism (Chiu 2002, 2010, 2017). The emergence of the Just Transition movement in the 1970s represents a continuation of the red–green negotiation and coexistence tradition (Gambhir et al. 2018; Atteridge and Strambo 2020). In August 2020, the Scottish Just Transition committee issued its Just Transition report, stating that job security and a friendly environment are not opposing goals (Pinker 2020). The last time Just Transition appeared was when Silent Spring ignited the environmental revolution, and the climate crisis has prompted it once more. Western industrial countries have dealt with the red–green competition before. For example, the European Union’s “European Green Deal (2021–2027),” unveiled in December 2019, emphasizes the establishment of a Just Transition mechanism (EU 2020; Heyen et al. 2020; Franssen and Holemans 2020).

Just Transition has recently focused on justice issues arising from the low-carbon transition, such as carbon taxes, the impact of energy prices on specific industries and vulnerable communities, coal miners for a long time in Europe, and economic vulnerability under carbon–neutral measures. On a case-by-case basis, coal mines have the most issues, followed by local renewable energy, transportation, and forestry, where the low-carbon transition is problematic (Wang and Lo 2022; Evans and Phelan 2016; Healy and Barry 2017; Heffron and McCauley 2018; Snell 2018; Schwanen 2020; Krawchenko and Gordon 2021).

Although there are many excellent papers on air pollution inequalities, most studies have focused on environmental justice and explored social inequalities (ethnicity, disadvantaged groups, and socioeconomic conditions), the spatial distribution of health impacts, and the distribution of high pollution sources (e.g., the petrochemical industry) concerning air pollution and health, rather than inequalities caused by policy interventions in governance—Just Transition (Perera 2018; Hill et al. 2019; Fairburn et al. 2019; Samoli et al. 2019; Fowlie et al. 2020; Kopas et al. 2020; Symanski et al. 2020; Pierangeli et al. 2020; Tessum et al. 2021; Davide et al. 2022; Gouveia et al. 2022; Mullen et al. 2022). In comparison, few studies have focused on air quality governance and Just Transition. For example, Shen et al. (2020) explored the link between air pollution and social inequality. They proposed using a social inclusion framework to address the disparities in the impact of air pollution on socially disadvantaged groups in rapidly industrializing countries. Meanwhile, Schroeder (2020) noted that the industrial restructuring for improving air pollution should ensure that people who are negatively affected do not lose their livelihoods as a result of the implementation of air pollution control measures.

Studies in Taiwan have explored air pollution and general environmental governance inequalities from an environmental justice perspective. Fan (2012, 2014, 2017) presented many local environmental justice studies in Taiwan, including crossregion aqueduct projects, wind turbine installation in Miaoli, and nuclear waste in Lan-Yu and agricultural land contamination in Qishan (Fan and Chiu 2019). According to Jobin (2020), disadvantaged areas face inequitable environmental and health damages from the Mailiao Refinery. Meanwhile, Shih and Tu (2017) stated that relying on a single source of information for policy decisions limits the role of the government and undermines decision-making legitimacy. Furthermore, they discuss how citizens in Taiwan have unequal access to general knowledge and participation in environmental regulation and scientific assessment decision-making (Shih and Tu 2019). Nonetheless, the research topic has been less focused on the Just Transition of air pollution and air product policy.

To summarize the literature review, more emphasis is being placed on Just Transitions in climate change research. Most literature focuses on or is intended for low or net-zero transitions; however, due to its definition and history, Just Transition may have broader implications for related issues such as air pollution. The genealogy of Just Transition research is extensive. According to the nature of justice, environmental and social justice frameworks divide it into three dimensions: (1) distributive, (2) procedural, and (3) recognition justice. To enrich its content, some scholars added a fourth, restorative justice, or another conceptual restructuring (McCauley and Heffron 2018; Pai et al. 2020; Wang and Lo 2021; Upham et al. 2022). Because the four types of justice differ in nature, we can divide the research into four dimensions to correctly identify and analyze the problem, deeply understand why a transition may advance or stall, and then propose a solution to the problem. The research takes as its object the HDDV protest movement affected by the air quality policy and employs a Just Transition framework based on environmental justice to contribute to the body of knowledge on Just Transition.

## Framework and methodology

### Research framework

**Fig. 1:**
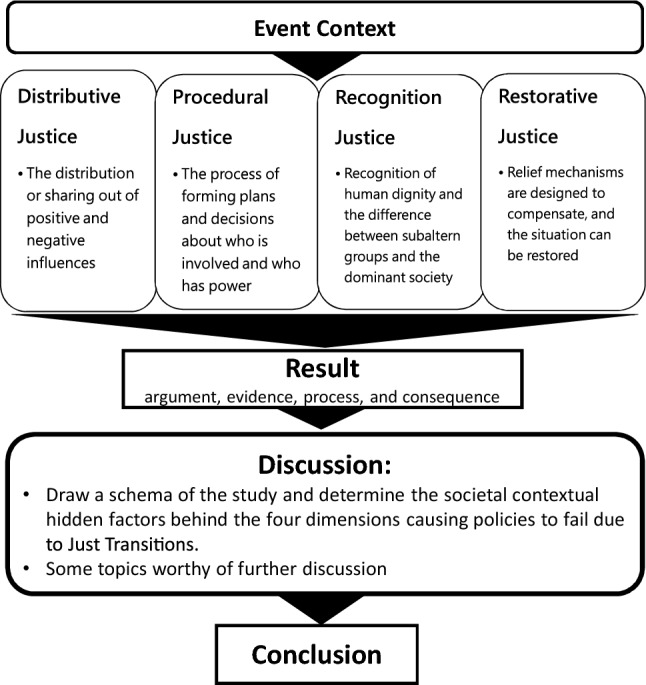
Research framework. Data Source: The authors, reference to Heffron and McCauley (2018) and Pai et al. (2020)

The research framework is based on Pai et al. (2020) to analyze Just Transition in depth and according to environmental and social justice. They employed the Just Transition analysis, which uses four dimensions of justice to examine basic environmental and social justice propositions. In classic justice research, distributive justice means sharing positive goods (resources, benefits, and health) and negative influences (harm and risks). Meanwhile, procedural justice involves making plans and decisions about who is involved and who has power (Schlosberg 2007; Fan 2017; Doorey 2017). Recognition justice recognizes human dignity and distinguishes subaltern groups from the dominant society. Its operational definition is to determine who is respected and who is not (Honneth 2004; Fan 2012; Walker 2012). Moreover, restorative justice is always required for the damage caused by a victim’s unpredictable and unavoidable injustice; relief mechanisms are designed to compensate and restore justice (Stark 2016; Braithwaite et al. 2019; Bratspies 2020; Pai et al. 2020). The framework of the four forms was adopted in the study (Fig. 1).

The present study separates the statements or arguments from the verbatim transcript in the results section based on the arguments, evidence, process, claim, and consequence. The results are important for finding the correlations and critical elements between the outcomes in addition to presenting the outcomes of the four dimensions (Walker 2012). The discussion section aims to find the key factor in a social context that causes policies to fail behind the superficial phenomenon, and then draw a study schema while reflexively considering how to deal with the Just Transition issues and some topics worthy of further discussion (Fig. 1).

### Methodology

This study used in-depth interviews and secondary data analysis as research methods. In-depth interviews examined actors’ descriptions, ideas, plans, or explanations of specific situations or events (Milena et al. 2008). Interviewees may share everything they know about the research topic during the interview. This study could extract much data from the information provided through interviews, which are purposeful conversations. By analyzing interview records, researchers can obtain longitudinal data from a spatial and temporal context in which the researcher is not personally involved. Researchers can then investigate and validate the data using theoretical and research structures (Boyce and Neale 2006).

Based on the characteristics of the research questions, the study employed the semistructured interview method, in which the interviewer asks questions according to an outline and invites 20 interviewees (23 times). Subsequently, the interviewer analyzes clues for follow-up questions from the interview’s verbatim transcript (Johnson 2001). Transportation business owners, drivers, Old Car Protest participants, environmental groups, officials, and scholars participated in this study. Table 1 shows the interview results. In addition, this study uses the secondary data analysis method, using news reports, official documents, or policy statements to analyze a relatively objective state of affairs (Table 2). Thus, the analysis process and results were credible and valid.

**Table 1:** List of interview data. Data source: Produced by the author

Type of interviewees	Code	Interviewee	Interview time/location	Interview format
Participant in the Old Car Protest	M1a M1b	Chief Convener of the Old Car Protest	November 25, 2018/Kaohsiung August 10, 2019/Kaohsiung	Semistructured in-Person Interview
	M2	Participant in the Old Car Protest	December 21, 2018/Kaohsiung	Semistructured in-Person Interview
	M3	Participant in the Old Car Protest Contact with Central Group	December 21, 2018/Kaohsiung	Semistructured in-Person Interview
	M4	Old Car Protesters in Chiayi	December 21, 2018/Kaohsiung	Semistructured in-Person Interview
	M5	Participant in the Old Car Protest Convenor of the North Group	April 21, 2019	Online semistructured in-Person Interview
	M6	Retired driver of old cars in the North	April 28, 2019/Taipei	Semistructured in-Person Interview
	M7	Participant in the Old Car Protest Convenor of the South Group	August 10, 2019/Kaohsiung	Semistructured in-Person Interview
	M8	Transportation industry operators, car dealership owners	January 14, 2020	Online Semistructured in-Person Interview
	M9	Transportation industry operator	August 12, 2020/Taichung	Semistructured in-Person Interview
Government	O1	Chief of the first level of central competent authority	December 19, 2016/Taipei	Semistructured in-Person Interview
	O2a O2b	Chief of the first level of central competent authority	May 5, 2017/Deputy Director’s Office of TEPA November 8, 2020 /Taipei (expert consultation meeting organized by a nonprofit organisation)	Semistructured in-Person Interview Semistructured in-Person Interview
	O3	Chief of central competent authority	December 25, 2019/Taipei	Semistructured in-Person Interview
Preowned car importers–exporters	I1	Transportation industry operator Preowned car importers–exporters	May 12, 2021	Online Semistructured in-Person Interview
Environmental groups	CG1	Senior leader in Environmental groups	December 14, 2016/Taipei	Semistructured in-Person Interview
	CG2	Senior leader in Environmental groups	September 30, 2020/Taipei (expert consultation meetings organised by the think tank)	Semistructured in-Person Interview
Scholar	S1	Scholar 1	March 12, 2014/(Agricultural Exhibition Hall, National Taiwan University)	Semistructured in-Person Interview
	S2	Scholar 2	October 26, 2016/Taipei (Conference Room 1, National Taiwan University)	Semistructured in-Person Interview
	S3	Scholar 3	April 7, 2017/Taipei	Semistructured in-Person Interview
	S4	Scholar 4	November 8, 2018/Tainan	Semistructured in-Person Interview
	S5a S5b	Scholar 5	July 29, 2021 August 27, 2021	Online Semistructured in-Person Interview (expert consultation meeting organized by a nonprofit organisation)

**Table 2:** List of official documents and policy statements Data source: produced by the author

Agency name	Title	Years
Executive Yuan	Press conference on explain the results of the post-election review of the Executive Yuan	2018
	Improved plan for pollution improvement of large diesel license plates, a meeting proposal of the academy (Transportation, Environment and Resources Division)	2019
Legislative Yuan	Legislative Yuan related documents, Yuan-Tzong-Tze-No. 970	2018
TEPA	Air pollution control action plan to reduce red alerts by half, report from director of Department of Air Quality Protection	2018
	General explanation of the amendment to the air pollution prevention and control law	2018
	EPA implementation performance and improvement measures of air pollution control action plan	2019
	TEPA’s operational report and legislative plan	2020
	Air pollution control plan (2020–2023)	2020
	Public hearing on ‘Progress and Review of the New Air Pollution Control Act’	2020
	Stellar achievements in old vehicle replacement. TEPA continues with support and improvement measures	2020
	Air quality standards	2021

^a^News reports are only listed in reference, but not included in this table

^b^All listed documents are also in the reference

## Analysis of the case and arguments

### Distributive Justice I: dispute on the fairness of accountability

Modern air quality governance shall be evidence-based. Air quality governance is based on choosing standards and tools based on each source’s impact on air quality and the cost-effectiveness of controls (O1 and S4). The principle is to prioritize high-impact rules and regulatory efficiency to ensure the effectiveness and fairness of accountability. However, regulations are more complex than scientific processes, and there is still uncertainty in the scientific assessment and the cost-effectiveness analysis of air quality governance. How elements are weighted in monetization calculations is also ambiguous (S1, S5a, O1; Farrow 2011; Pennell 2011; Hidy et al. 2011). Therefore, the fairness of policy interventions is a significant argument for distributive justice.

In 2017, stationary sources accounted for 27–31% of the scientific evidence available to the TEPA through model-3/CMAQ, mobile sources accounted for 30–37%, and other pollution sources accounted for 32–43%. Furthermore, HDDVs contributed 18.3–24.8% of the source of PM2.5 and NOx pollution, which is significantly higher than petrochemical, steel, and power plants (Wu et al. 2017; TEPA 2017, 2018a, 2020d). Until 2019, the TEPA continued to recommend prioritizing HDDV control over other pollution sources (Wu et al. 2020; S5a; TEPA 2019, 2020a). Therefore, the TEPA’s plan to promote air quality control identified improving HDDV control as the most critical measure (O1). However, some academics argue that the mainstream approach undervalues stationary sources (S2, S3). In December 2016, a distinguished environmental engineering professor argued on public television that in some areas of Taiwan with high stationary source emissions, PM 2.5 emissions from power plants and factories were underestimated by 15.2%. The total stationary source PM2.5 (primary and secondary formation) should increase by 1.7 times, whereas HDDVs should decrease by 30% (S3; Public Television Service 2016). The TEPA, environmental protection groups, and experts all agreed to address the disagreements. After an internal meeting with experts from two different advocates, the TEPA adopted a more compatible approach: keep the original proposal but increase stationary source control measures (O2a). However, environmental protection groups and NOCSA still worry about accountability due to the (contentious) scientific assessment (CG1; CG2; O2b; S5b).

### Distributive justice II: fairness of cost differences for car replacement

The second important dimension of distributive justice is the fairness of the cost difference for car replacement in the transportation industry between HDDV business owners, employed drivers, and license-leasing drivers. Depending on the category, establishing a transportation business in Taiwan requires capital ranging from US$1 to US$3.3 million. Companies have many self-employed transportation drivers because transportation companies do not employ enough drivers to meet market demand. To legally engage in transportation work, these drivers must register their license plates in the name of the car dealership and pay the company a monthly license-leasing fee of $66–$133. The license-leasing drivers are in charge of the vehicle’s safety, loans, and maintenance, which is the foundation of license-leasing (Huang 2003; Yang 2016). Approximately 70% of HDDVs in Taiwan are license-leasing drivers (M6; Wang 2004; Ministry of Transportation and Communications 2020a, b). A car dealership and transportation business owner who participated in the protest (M8) said:

In fact, the impact on we owners are not significant. I have 28–38 registered vehicles myself, and having subsidies for car replacement is cost-effective for me. Business owners would regularly purchase new cars and sell old cars. Many dealerships are buying new cars, so the regulations promote the purchase of new cars, and we can receive subsidies…

In the initial plan, self-employed license-leasing drivers could receive up to approximately US$16,700 per vehicle, but only for new cars, not used ones. Converting a new HDDT into a vehicle costs US$50,000–66,000 (e.g., converting to a tow truck). Self-employed license-leasing drivers could sell their old car for up to US$33,000 and then buy a new one (M3). Due to the new Act, selling old cars in Taiwan was impossible. Car replacement will leave drivers in debt, no matter how good the loan. M8 said:

Following the ban on Stage I–II diesel cars, the old car drivers are worried that their old cars cannot be sold, in which case, the overall burden (to buy a new car) would surpass the drivers’ affordability. Usually, drivers sell their old cars to have enough money to buy a new one. Due to the ban on Stage I–II diesel cars, old cars can only be sold overseas, but we, old car drivers, do not know how to sell vehicles abroad, and the government has also not made it clear.

Another member of the IOC-Protest (M7) said:

It is a matter of the right to work and live. There was no way to sell the old car after it was banned. The loan on the old car is still not paid off, and we must take out another NT$2 million loan to buy a new car (which equals approximately US$66,000), making life difficult for our family.

A preowned HDDV dealer for 11 years (I1) said:

At first, we, the preowned car exporters, did not know about this (the government’s control of Stage I–II diesel cars). The car dealerships only informed us about this when the protest started… in the past, drivers did not sell the vehicles directly to us (importers and exporters), but to the large transportation companies…there was about a year in between when HDDV drivers thought that old cars could not be sold and had to be scrapped. The drivers had nothing left and could not survive.

Aligning with old car drivers benefits both parties for car dealership owners. In the face of increased regulations and the phase out of old cars, large transportation companies and license-leasing drivers have unequal tolerance. The transportation business owners can benefit from the subsidies because they can use the money to pay for regular vehicle replacements and save money, while the employed drivers suffer no loss. Meanwhile, forced car replacement may cost license-leasing drivers around $33,000 in losses or debt. NOSCA questioned whether the government considered the cost differences' fairness in car replacement.

### Procedural Justice: license-leasing drivers and the preowned car industry are left out of the decision-making process

Citizen participation in 2017 environmental policy was a great reform outcome. This was meant to increase citizen process in policymaking. During its decision-making process, the TEPA consulted environmental professionals and affected business practitioners first, then anti-air pollution communities and civic groups. Why did the Air Pollution Control Act amendment ignore license-leasing drivers if the new Act to tighten HDDV control and the compensatory measures are contentious in terms of distribution justice? They will be most affected by the Act. A chief officer (O2b) stated:

After I took over in 2016, we made plenty of efforts to include citizen participation in the decision-making process. We could find civil stakeholders for stationary source discussion; they were people who had been engaged in the environmental movement for a long time so they could advise on the policies within the system. But for old diesel cars and two-stroke motorcycles, we could only find industry associations. We did not know of any civil society group…indeed, we left out some people with whom we should have communicated.

In addition, a transportation practitioner (I1) mentioned:

In the early days, the TEPA approached local, industry-specific transportation associations rather than industry groups. Due to the prevalence of ‘license-leasing’ in Taiwan’s transportation industry, the situations of associations, small and medium-sized business owners, and license-leasing drivers varied greatly….. Small- and medium-sized business owners and license-leasing drivers did not know what the associations were doing even if they joined. Large business owners operate these associations. Therefore, the government did not understand license-leasing drivers’ position.

Meanwhile, the official responsible for the control of the transport industry (O3) stated:

When amending the Air Pollution Control Act, the TEPA followed our standard procedure of looking for environmental protection groups, advocates and industries with whom they were familiar. In terms of mobile sources, they consulted transportation business owners, who represented only part of the entire transportation industry, and they did ignore the communication with two-stroke motorcycle and Stage I–II diesel car drivers, who participated in the protest at the initial stage… Indeed, this is not a purely scientific and technical issue but a complex issue of communication and negotiation.

Preowned car dealers stated (I1):

It was not until April 2019 (actually, it was finalized in March) that the government stipulated that there could be a maximum subsidy for purchasing used qualified HDDVs. Before this, many old car drivers were very concerned that their old cars could only be scrapped, forcing them to buy new cars and thus incur increased debt.

Initially, large transportation companies with capital did not contact or collaborate with preowned car importers-exporters, instead negotiating with the government for greater benefits for themselves. During the Air Pollution Control Act amendment, the government sought industry groups through large business-led associations and approached anti-air pollution groups for civil matter communications. Anti-air pollution activists were more concerned about emissions from factories and power plants and had little to say about the tightened controls on HDDVs. Furthermore, the government failed to communicate effectively with the used car industry. Assume that the government’s assistance measures encouraged the export of phased-out I–II HDDVs and opened up subsidies for using vehicles. In that case, old HDDT drivers may not have felt compelled to protest due to their concern for their families’ livelihoods (M5, M8).

### Recognition Justice I: sense of disrespect and stigmatization

In this section, the concept of Recognition Justice will be explored. A significant portion of environmental justice research emphasizes quantifiable losses and the subsequent methods of compensation, with calculations for losses and compensations grounded in scientific evidence, such as the health detriments resulting from air pollution. However, in the context of a Just Transition and the environmental public policies, it is necessary to partially shift the focus from exclusively objective, measurable factors to the more subjective elements, including individuals' value judgments, beliefs, and self-identification, which prove challenging to assess objectively. It is critical to understand that a transportation industry protest is equivalent to a strike, during which drivers are not paid (M1b, M7, M9). They have no access to social relief channels for their losses during the movement, regardless of subsequent relief. They protested because they felt disrespected. A movement leader who previously worked as a transportation contractor for a large petrochemical industrial zone (M1b) said:

I have seen those giant smokestacks in the petrochemical industry. I looked up and saw that the factories were emitting vast amounts of exhaust fumes while the government was forcing us to replace our cars to meet the new standards (that is) the diesel cars that we depend on for our livelihood. This is unacceptable.

NOCSA comprises HDDT drivers from the South and Central regions, all of whom have previous experience working for large plants. Although they do not understand the scientific data, how can the “little smokestacks” in their vehicles outperform the giant smokestacks that never sleep? Although the large factories are financially powerful, the government forced the drivers to trade in their vehicles, creating a sense of disrespect. When NOCSA members first learned about the enhanced control of HDDVs, their first thought was that the government’s policy labeled them as “polluters.” This is why they yelled, “Innocent the old cars,” because their underlying message was, “DO NOT STIGMATIZE US, WE ARE NOT DIRTY”. They wanted to understand why elderly drivers should pay for “better air quality.” An old car protest convenor (M1a) said:

The drivers certainly know that their HDDTs create pollution, but we have always believed that the big chimneys (from the factories) emit more pollutants. The government cannot regulate the big factories, so the focus was shifted to us, and we were made responsible for air pollution. We want to shout out loud that “innocent the old cars.”

A transportation business owner M8 said:

They said, “Why do cars become illegal and pollute the air when they are old? Why cannot old cars that were once legal be on the road again? We regularly inspect our vehicles by the law, and now I’m told I cannot drive my car because it is old and creates pollutants.” This made them uncomfortable. The main target of policy communication is only large companies and their owners, so I felt disrespected.

Many NOSCA members have worked in the transportation industry in large petrochemical plants. They wonder why the government does not dare to control large enterprises’ big smokestacks but manages their small smokestacks. They also discovered many academic studies (related to distributive justice) in which the authorities overestimated the transportation industry’s emissions, so they felt stigmatized. The motorists began to inquire about anti-air pollution and anti-pollution policies. Later, they discovered that among environmental protection groups, there have always been advocates of “hunting the smaller one while ignoring the larger,” and they believed that these advocates could destigmatize them.

### Recognition Justice II: citizen’s perception of air pollution

The civil anti-air pollution movement that emerged in 2010 grew out of the experience of combating stationary sources (stationary sources of air pollution), which saw giant smokestacks and factories as the primary source of air pollution (CG1; Walther and Chou 2018). A civil nonprofit organization leader engaged in the environmental protection movement for more than 20 years (CG2) stated:

We certainly recognize that transportation can also create air pollution, but various transportation control standards have been tightened continuously for decades...on the contrary, large factories were punished only with small fines even though they have many violations of emissions…air pollution is already known to be serious, but air pollution charges have not been raised enough to make factories change their emission behavior so that our air quality can meet WHO standards.

Polls on air pollution from 2016 to 2020 revealed that roughly 60–80% of the public regarded air quality as poor, putting political pressure on the government. As a result, following the change of political parties in 2016, the ruling DPP made improving air quality a priority policy (O2a). In 2017, the TEPA expanded its knowledge database and increased public participation in air quality improvement by appointing more academics and environmental or anti-air pollution activists to various committees. This has gradually increased public awareness of the dangers of air pollution (O2a).

The 2018 referendum, held concurrently with national and local elections, is of great political significance, and the impact of air pollution issues on politics has peaked (Chen 2018). Three of 10 ballot proposals addressed factory or power plant air pollution, and all three passed (Central Election Commission 2018). This shows support for better air quality. Anti-pollution groups and NOCSA argue the government over-regulates mobile sources in the transportation industry and area sources like restaurants and ground dust (CG1; CG2; O2b; S5b). The perception of fairness in enhancing regulation accountability, inadequate communication about the decision-making process and the feelings of old car drivers, and the anti-air pollution movement’s perception of “hunting the smaller one while ignoring the larger” all exerted political pressure on the government, despite high consensus for air quality improvement. Therefore, recognition justice, which cost the ruling party the November 2018 local election, influenced subsequent events.

### Restorative Justice: legitimate expectations

A movement member who is a legal professional (M2) argued that enforcing administrative sanctions of mandatory phase out by law would violate legitimate expectation. Environmental regulations can be improved over time, but usually not retroactively. Thus, the government compensation for increased controls should be based on maintaining legitimate expectations rather than administrative incentives (M2). According to the calculations in the preceding sections, each license-leasing driver may lose or be in debt of approximately US$33,000 following the implementation of the new Act. Even if the government offers them preferential loans, the drivers may still be in debt (M3, M4). Just Transition advocates that the government ensure that all stakeholders’ claims are protected (Gambhir et al. 2018). As a result, Tasini (2021) proposes a high standard Just Transition, with the amount of restoration ensuring that the affected labor or family retains 100% of their original income. The reasoning is that society, which previously benefited from fossil fuels, should bear the cost of the transition rather than specific industry workers.

Since August 16, 2017, the government has repeatedly strengthened restorative justice measures to address public discontent and the political crisis caused by NOCSA. The new TEPA administrator stated on March 8, 2018, that as long as the vehicles met the Stage IV standard, the target of phasing out 86,386 vehicles could be reduced to approximately 20,000 vehicles to reduce the impact. In addition to subsidizing the purchase of new and used cars, the new policy includes tax breaks and other benefits, bringing the total to around $33,000 and extending the subsidy period until December 10, 2021. (Executive Yuan 2019; Liu 2019; Sun 2020). In the case of being able to sell the old cars, license-leasing drivers can maintain their livelihoods (losses and liabilities of approximately US$16,000 or less) after replacing their cars. In March 2020, the subsidy period was extended to 2022 due to the impact of COVID-19 (TEPA 2020b). To summarize, NOCSA stated that government compensation should be based on upholding legitimate expectations rather than administrative incentives. Second, they insisted that the government be held liable for damages while fully recovering their losses (covering the entire cost of a new car), rather than providing additional benefits to vested stakeholders.

## Results

This article presents six arguments based on literature and interviews. The results were grouped by argument, evidence, process, claim, and consequence (Table 3). The study found that TEPA formulated an article to improve the regulation on HDDVs older than 10 years and expects to accelerate the phase out of old vehicles through car replacement subsidies. The government’s decision-making process was missing license-leasing drivers and preowned car dealers, resulting in an insufficient replacement budget for old car drivers and their inability to trade in preowned cars. However, license-leasing drivers were initially unaware that old cars could still be sold to preowned car dealers. As a result, they believed trading in their cars would jeopardize their livelihood. The policy may benefit large dealership owners. Due to new regulations, HDDVs can no longer be driven. Under legitimate expectation, drivers’ losses caused by the policy should be compensated.

**Table 3:** Compilation of research results. Source: Authors’ own work

Concepts	Argument	Evidence	Process	Claim and consequence
Distribution Justice	Strengthening the fairness of accountability between the factories and transportation industry	According to the TEPA’s method, HDDV emission accounts for 18.3–24.8% of pollution. However, different studies have also suggested that the HDDT contribution is 30% less than the officially cited method	The civil antiair pollution movement stems from the fight against factory and power plant emissions and their distrust of government regulation of stationary sources	Antiair pollution groups and NOCSA advocate that the government hunts smaller vehicles while ignoring larger ones, causing unfair accountability The TEPA invited experts to an internal meeting, and although no consensus was reached, the control of stationary sources was enhanced The estimation methods and results used by the TEPA have remained the same
	Fairness in the cost and affordability of car replacement	Drivers receive up to US$16,000 worth of subsidies for car replacement. However, a new car will cost US$50–$66 thousand. The old car still has a value of approximately US$33 thousand	Owners or operators of the car dealership can even profit from the replacement of cars. License-leasing drivers lose or are indebted to US$33 thousand	NOCSA advocates that the replacement of vehicles affects their livelihood. The government raised the subsidy to a maximum of approximately US$33 thousand. However, drivers later learned that there were channels to export stage I and II cars
Procedural Justice	The voice of key stakeholders is missing in the decision-making process	The government looked for large business operators in the industry, sought antiair pollution groups as civil stakeholders and did not reach out to license-leasing drivers or preowned car dealers	The government should have communicated with license-leasing drivers. The lack of information, such as not knowing that stage I and II cars, can be sold, caused panic among old car drivers	NOCSA asserts that the lack of a process has left the decision-making process devoid of measures to protect the rights of license-leasing drivers It was not until NOCSA protested that the TEPA learned of the arguments that license-leasing drivers should have made and used car dealers in decision-making
Recognition Justice	Sense of relative deprivation, stigmatization	Many NOCSA drivers have worked for bid factories and have seen those giant smokestacks emitting pollution 24/7. They also know how rich the big businesses are	HDDT drivers’ experiences create a sense of relative deprivation and stigmatization among them	They advocated the innocence and non-stigmatization of old cars. Close to the election period, the IOC Protest resulted in the election defeat The government ended its policy of accelerating the phase-out of HDDVs that are over 10 years old
	Citizen perceptions influencing policy	According to multiple surveys from 2016 to 2020, 60–80% of the public believed that air quality was poor. The antiair pollution movement predominantly fights against a power plant and factory emissions	The antiair pollution movement and IOC Protest argue that the ‘hunting of the smaller vehicles while ignoring the larger vehicles’ argument has influenced the public’s general perception	
Restorative Justice	Legitimate expectation	The new regulations predominantly affect Stages I and II HDDTs, followed by stage III cars	HDDTs over 10 years old are legal. If they are to be phased out, the government should refrain from measuring the incentive mechanism's compensation amount	NOCSA argues that the government should adopt a higher standard of compensation based on the principle of legitimate expectation The government first announced that HDDTs were only required to meet the stage IV criteria; later, the old cars were no longer forced to be replaced with new ones. The subsidy was raised to a maximum of approximately US$33 thousand. The livelihood of driving can be maintained

Disrespect and stigmatization drove NOCSA to protest. The drivers thought the government’s treatment of large, financially powerful factories that emit 24/7 and have small smokestacks was unfair based on their factory experience. The policy also labeled them “polluters.” In a context where the public was dissatisfied with air quality, the movement was more concerned about factory and power plant emissions, and the discourse that the government was “hunting the smaller one while ignoring the larger” was voiced in society. Air pollution and the IOC-Protest increased political pressure, bringing down the ruling party. Eventually, the government terminated the policy.

The results indicate that license-leasing labor relations are a critical social contextual factor in Taiwan’s transportation industry, and the results of the four dimensions of the analysis are directly or indirectly related to license-leasing drivers. The cause of this case is that license-leasing drivers are ignored in communication; due to improper procedures, decision-makers disregarded license-leasing labor relations, resulting in a lack of consideration for the loss of license-leasing drivers and the difference in subsidy benefits. The aforementioned distributive justice and procedural justice have an impact on the feelings of license-leasing drivers, who felt disrespected and stigmatized; as a result, they can only rely on the principle of legitimate expectations, make the most positive claims, and fight to defend their rights and interests. They eventually rose up to fight, resulting in the policy’s failure.

## Discussion

### The schema of the study and policy implications

**Fig. 2:**
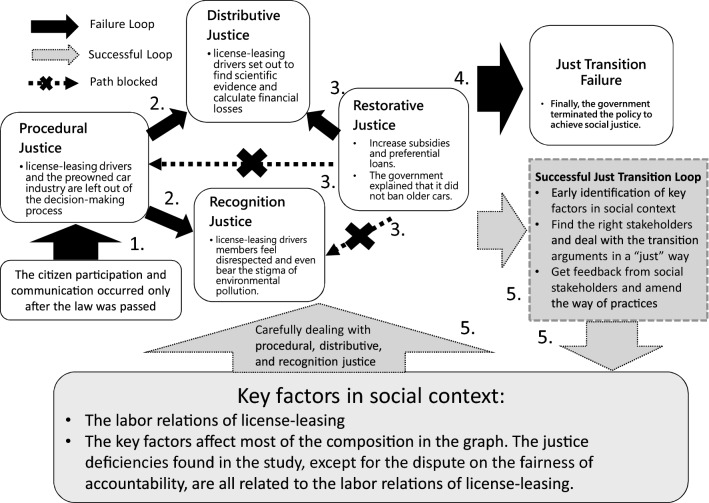
The schema of the case. Data Source: The authors

The study’s results can be represented as a schema (Fig. 2). First, this schema could be used to analyze other Just Transition cases in Taiwan or other countries for reference and comparison purposes. Second, policymakers can use the schema’s policy implications to develop future environmental policies to avoid becoming trapped in a Just Transition problem and being unable to move forward.

The inequity of gains and losses (distributive justice) is frequently the starting point in some environmental and social injustice cases. However, the real cause of the incident was that these drivers only found out about the passage of the regulation on large diesel vehicles six months after the law had been passed. The decision-making process lacked procedural justice, and citizen participation and communication occurred only after the law was passed (1). Injustice sparked the conflict. Then drivers who leased their licenses began to find scientific evidence and calculate financial losses (2). They feared the new decree would affect their future right to work and live, raising issues of distributive justice. Then, license-leasing drivers believed the government communicated with large car dealers but not with motorists and tightened control over large diesel vehicles but not large factories (these are drivers’ feelings, not facts), so they felt disrespected and took on the stigma of causing environmental pollution (recognition justice) (2). Government increased subsidies and explained policies. The government missed the best chance for procedural justice and did not address the disrespect and stigma of license-leasing drivers (3). The situation deteriorated to the extent that NOCSA fought back vehemently, defended legitimate expectations, demanded destigmatization, and expressed its rage through voting behavior, which had a huge impact. As a result, only social justice was met, and Just Transition governance failed (4). If the government addresses the key social factor while building the “Successful Just Transition Loop,” the results should be different (5). The loop begins with identifying stakeholders and inequities, then carefully dealing with procedural, distributive, and recognition justice, and finally receiving feedback from social stakeholders and amending practices.

### The red–green context in Taiwan

The Yellow Vests movement and the IOC-Protest are declarations that environmental policy should aim for a Just Transition. It does not need to reach the ideal heights of Green Marxism, nor does it have the same political ambitions as the red–green synthesis, but it requires consultations that address justice issues to succeed.

In 2022, Taiwan is creating a plan to achieve carbon neutrality by 2050, joining the prosperous Western countries in the rapid low-carbon transition. Taiwan does not have a coal mining industry and thus escaped the first wave of the global Just Transition test. However, it is already confronted with the issue of developing agricultural land using renewable energy and competing for fishing rights. A significant upcoming challenge for Taiwan will be balancing the acceleration of the low-carbon transition with adequate work to address the transition of energy-intensive industries, which account for approximately 30% of total carbon emissions (Chou et al. 2019; UNFCCC 2020; Chou and Walther 2022). In recent years, the environmental movement has developed to the point where it can influence and promote policy (Chou 2015). The impact of civil society on the government is visible in Taiwan’s current air quality and low-carbon transition policy. However, economic developmentalism and anticommunist political repression influenced Taiwan. As a result, leftist and trade union forces are weak, and the labor movement’s development is limited (Liu 2008; Ho 2008; Chen 2019; Wang and Shieh 2021; Chen 2021). With such a social backdrop, Taiwan’s transportation industry is so prevalent in license-leasing labor relations, which rely on private trust rather than legal protection. Labor and society in the United States or wealthy Western European countries can hardly tolerate such labor–capital relations, whereas Taiwan has widespread exploitation with an incomplete labor–capital system; this point deserves special attention in the research and practice of Just Transition in Taiwan and particularly in East Asian countries.

The balance of environmental and labor rights is so skewed in favor of sustainability that, although the TEPA approached environmental groups, it ignored grassroots license-leasing drivers and preowned car dealers. This echoes Walther and Chou’s (2018) argument that the government lacks crossfield risk governance capacity and focuses on engineering expert networks with little involvement in social, legal, and cultural networks. Furthermore, according to its analysis of the air quality decision-making process, institutional TEPA lacks many experts and citizens in the design of third-party supervision and participation, resulting in a “blind area of environmental governance” (Walther 2018). This blind spot will result in boundary work (the phenomenon of excluding dissidents), which confines decision-makers to small circles of limited shared knowledge networks. Because the government is unfamiliar with social grassroots networks, it may result in biased decisions. NOCSA’s claim is supported by license-leasing drivers who believe environmental policies undermine their rights. The research on green and red competition should be expanded, and the core issues of Just Transition should be thoroughly examined to promote sustainable development in Taiwan based on fairness and justice (Chiu 2002, 2010, 2017). The government’s poor handling of Just Transition is the worst-case scenario for the people. Environmental policies are hampered by Taiwan’s democratic system, which allows disgruntled groups to express their concerns and even protest in front of the Presidential Palace.

### The factors of subjectivity in Just Transition

The article finds important subjectivity factors in Just Transition, such as proper communication procedures to avoid stigma and disrespect. Tsoi et al. (2021) found that communication problems and monetary incentives are insufficient to eliminate HDDVs. Actively eliminating HDDVs challenges citizen engagement. The fairness of cost differences for car replacement observed in this study may find clues from the California transportation transformation program to protect workers from disproportionately bearing the cost of cleaner trucks (Zabin and MacGillvary 2020). This study proposes that legitimate expectations should be based on restorative justice, consistent with Zabin and MacGillvary’s (2020) recommendations for the trucking industry’s demand-side workforce policy levers for job quality and supply-side workforce development strategies.

Like current climate policy, environmental governance, and sustainable transition, air quality governance will reach a point where Just Transition and institutional design are carefully considered. At this point, the focus of climate policy and environmental governance is not only on nature, but also on people and the environment as a whole (Barry 2005; Gross 2014). Because Just Transition is human-centered, exists in a complex social context, and involves intellectual debate and political mediation, recognition justice may be the most difficult aspect of Just Transition (O2a). Therefore, the low-carbon transition and air quality policies must be carried out with equity and justice (Eisenberg 2019). It is appropriate to examine the various arguments for Just Transition using various concepts of justice (Walker 2012; Gross 2014); that is, the framework proposed by Pai et al. could be used appropriately to facilitate Just Transition case studies. Specifically, two of the Just Transition themes mentioned by Wang and Lo (2021), Just Transition as a labor-oriented concept and Just Transition as public perception, correspond precisely to the Just Transition scenario described in this study. This paper expands upon the air quality issue currently lacking in Just Transition research.

### The consequence of nonforced phase out

What is the impact of the policy change in 2019 to a strategy of voluntary phase out, lenient determination and encouragement of car replacement? Table 4 highlights that according to the data of March 2022, there is still 73,655 to 79,769 stages I–III HDDTs over 15–16 years old and 46,290 to 50,472 stages I–II HDDTs over 23 years old (Ministry of Transportation and Communications 2020a, b). By March 2022, stages I–II vehicles declined by only 24–28%, whereas stages I–III vehicles declined by 36–40% (Ministry of Transportation and Communications 2022). The original 2017 air pollution control plan was to achieve an annual average PM2.5 concentration of 15 μg/m^3^ in 2020, whereas the new air pollution control plan in 2019 has postponed the target to reaching 15 μg/m^3^ in 2023. In addition, the newly announced PM2.5 concentration standard for 2021 was maintained at 15 μg/m^3^, which remained unrevised for nearly 10 years (TEPA 2017, 2020c, 2021) (Table 4).

**Table 4:** Quantitative results after imposing enhanced regulations for HDDTs. Source: TEPA (2020d, e) and Ministry of Transportation and Communications (2022), Table produced by the author

	Stage I–II Cars older than 17 years	Stage III Cars that are 11–17 years old	Total
2017 Target	86,386 (51.8%)	35,123 (21%)	121,509 (72.8%)
2019 Target	These emissions are sufficient to meet stage IV standards. Therefore, only 20,000 vehicles need to be phased out. The remainder will be regulated with incentives		20,000
Actual situation in 2022 171,483 HDDTs	There are still 46,290 to 50,472 vehicles aged 22–23 years or older	There are still 27,365 to 29,297 vehicles aged 15–16 years or older	Between 73,655 and 79,769
Percentage in 2022	27–29% (22–23 + years)	16–17% (16–22 years)	43–47% (16 + years)
Percentage of decline in 2022	24–28%	19–24%	36–40%

Based on a scientific assessment, the number of phased-out HDDVs was estimated to meet the desired air quality objectives in 2017. Regardless of the current policy shift, TEPA has never revised the scientific assessment decision made at the time (S4, O2b; TEPA 2020d, 2021). Due to political pressure, the termination of the enhanced regulation on HDDVs has slowed progress toward improving air quality. We can expect better quality in 2022 if the law is implemented in accordance with the 2017 Air Pollution Control Act. The improved control of HDDVs was a failure in terms of environmental management effectiveness. However, Taiwanese society has learned an important lesson: Just Transition must be handled cautiously.

Finally, some groups in the respondent pool are limited because there are few interviewee experts in air quality environmental engineering with modeling techniques, so the argument may be biased toward citizens’ subjective perceptions.

## Conclusions

Because of the global climate crisis, the importance of Just Transition has grown in recent years. When studying communities and low-carbon transition policies, CEE began to embrace Just Transition to ensure that issues of social equality and justice do not hamper environmental policies. There are currently more Just Transition cases for coal mines, renewable energy (land use), and fossil fuel power plants. However, it can be seen from the 2018 Yellow Vests movement and the movement that occurred in Taiwan the same year that the transportation industry’s campaign to defend its rights against the environment significantly influenced Just Transition. The increased pollution emission standards for vehicles manufactured 10 years or more ago and the phase out of diesel vehicles 10 years or older sparked a massive protest by the NOCSA, which Stages I–II old car drivers formed.

The antiair pollution campaign and some scientific controversies have raised concerns about factory and power plant controls and diesel vehicle controls, calling the levy’s fairness into question. Transport business owners can receive vehicle replacement subsidies, and drivers under care incur no losses, but license-leasing drivers face $1 million in losses or loans, creating an unfairness issue. The policymaking process excluded license-leasing drivers and used car dealers, whose roles and rights are not reflected. HDDT drivers’ disdain and stigmatization were enough to defeat the ruling party. Officials ended the policy after admitting IOC-Protest caused the loss. The shift to more substantial incentives has slowed progress in improving air quality, with 50,000 Stage I and II HDDTs still in use in 2022, 22–23 years or more, a 24–28% reduction from 2016.

The results indicate that license-leasing labor relations are a critical social contextual factor in Taiwan’s transportation industry, and the results are related to license-leasing drivers. If the government can manage the key factor in the social context and make appropriate arrangements for justice dimensions while constructing a successful Just Transition Loop, the results may be different. While previous research has primarily explored on the equity of air pollution risks, health, and socioeconomic conditions, this study’s air quality policy research focuses on the equity of its accountability and consequences, which can supplement existing Just Transition research. Furthermore, this study finds that Just Transition arguments are critical to the success of Taiwanese air quality governance, and that environmental policies require a cautious approach to Just Transition arguments to avoid failure.

## Data Availability

Every data are available on-line and were well cited.
